# Psora*This essay upon Psora was written by S. L. G. Leggett as a candidate for the Miller Prize. Our readers may remember a former mention of this prize. Dr. R. Gibson Miller, of Glasgow, came over to study Homœopathy with Dr. Kent. He was so well pleased with Dr. Kent’s lectures on the *Organon* that he left a fund for the establishing of this prize to be given yearly to the student who passed the best examination on homœopathic philosophy, etc.—Eds.

**Published:** 1888-04

**Authors:** S. L. G. Leggett


### HOMŒOPATHIC PHYSICIAN,

A MONTHLY JOURNAL OF MEDICAL SCIENCE.

"If our school ever gives up the strict inductive method of Hahnemann, we are lost, and deserve only to be mentioned as a caricature in the history of medicine."—CONSTANTINE HERING.

Vol. VIII. APRIL, 1888. No. 4.

### PSORA*

Is that miasm which forms the basis of the disease called itch, and of which miasm itch or eczematous diseases are the most favorable and the simplest manifestation.

This miasm may have been handed down from generation to generation, or may have been acquired through suppression of some of its forms of manifestation in this generation. It is hard to say in each individual case whence it came or whither it will go, its presence being the palpable fact with which *we have to deal,* leaving the *cause,* as we do in all physical phenomena, as light, heat, electricity, to be determined inductively from its *effects.*

This latent power, now so universal that it is a habitant of nearly every organism, may not in each individual case have exhibited an eczema—the *simplest form* of which, and the most easily cured, are the *itch vesicles upon the hands,* as the most *violent form* is the *squamous upon the head*—although most likely to have done so at some time; having once so appeared, and having disappeared or been suppressed, it ceases to be *latent,* becomes roused and *active,* all future manifestations being

* This essay upon Psora was written by S. L. G. Leggett as a candidate for the Miller Prize. Our readers may remember a former mention of this prize. Dr. R. Gibson Miller, of Glasgow, came over to study Homœopathy with Dr. Kent. He was so well pleased with Dr. Kent's lectures on the *Organon* that he left a fund for the establishing of this prize to be given yearly to the student who passed the best examination on homœopathic philosophy, etc.—EDS.

in line of progression and with increased destruction to the organic structure until the life of the patient ceases.

Old medicine, more conscientious than modern, had discovered, before the revered Hahnemann's time, that a suppression of eruptions; in other words, of psoric manifestations taking that form, was followed by direct consequences to the individual, according to his temperament (Juncker), and that no power they possessed could arrest the progress of that internal disease, any more than that they had the power to cure the eczematous manifestation by any use of external or internal remedies then known without *suppressing* it.

Conscientiously they recorded their efforts and observations in trying to remove these evils and their consequences. Yet modern, scientific (?) medicine does not seem cognizant of the fact, and goes blundering on in its perpetual, well-worn round, hoping to discover in some unheard-of region, by the aid of the microscope (!), forsooth, the *cause* of all the ills of all mankind.

Finding that suppression of the outward manifestation of latent power, the vesicle, tends to a deeper, broader, more active inroad of the real disease power upon the human system, we look for its more immediate effects, and find sometimes an acute attack that ends the life of the victim within a short time, again, a chronic and ever-growing miasm, shifting from the more superficial and distant organs to the more central and vital, until death comes to the rescue and relieves the suffering.

There may be acute or chronic lung disease, dysentery, diarrhœa, diabetes, hysteria, epilepsy, apoplexy, cancer, malignant growths, tumors, hydrocephalus, and effusions of the cavities, general dropsy, etc., until the list at first glance seems to cover all the diseases known to man.

After close observation of the phenomena of disease following the suppression of eruptions, Hahnemann, by inductive reasoning from the *effects* to the *same common cause;* by observation of the effects of the seemingly similar remedy, in cases where the remedy did not prove broad enough to cover the true diseased condition, but removed a few of the symptoms, only to have them again return, or new ones take their place, wisely concluded that this now *active* psoric miasm could not be covered in its entirety by the single remedy, as when in its eruptive stage; that it had so increased in its extent, expressions, and ramifications that it would usually present successive symptom pictures, and must be met by the similar drug pictures, until the whole disturbance—always supposing it to be curable—was eradicated.

Latent psora shows itself in the peculiar sensitiveness of the system to changes—climatic, dietetic, physiological, or psychological.

A system with a tendency to colds in every change of the weather shows the presence of this latent miasm; as a person in a perfectly healthy condition would not suffer sickness from so slight a cause, although the change might be disagreeable.

A system that is disturbed by the slightest excess at table in either eating or drinking also shows the existence of this mighty miasm. If there were perfect balance of the vital force, the appetite would not be in excess of the amount needed by the system, and the system would have assimilated the amount taken and have been strengthened by it, as at one time there might be a more extensive call for and use of the vital fluids of the system than at another.

Sickness should not follow a slight excess and gratification of sexual desire, but should be met by a corresponding recuperative ability on the part of the vital force. It is reasonable and a fact that throughout nature we always find a reserve force, an overplus of space, an overplus of strength, an overplus of motility, an ability to fit ourselves to surrounding circumstances that is seldom called for, but always found when needed. So we find that somewhere more than sufficient food is grown for the necessary supply, more than sufficient flowers blossom to give us delight—in fact, more than sufficient supply for all our necessities and pleasures; just as truly do we find an excess of vital force to meet an occasional inordinate demand. We are not speaking now of habitual abuse; there are those who are made ill by just these slight deviations, and it points clearly to the psoric miasm.

A great sorrow, loss, or trouble, is of far greater importance to the system than any of the above-mentioned disturbances—is slower of recovery, more lasting, and changeful in its effects; yet a healthy organism will show less of disturbance, recover more quickly and wholly from its deleterious effects, and the system sooner returns to its normal condition.

Should the *trial* be constant, the effort of the vital force to bear with, or to carry this extra burden will in time weaken and shatter the healthiest organism.

We frequently find in persons afflicted with psora an inanition of the whole or a part of the nutritive principles necessary for the healthy animal organism, or too many find an over-active assimilation or appropriation of one substance or more, at a great expenditure of strength to the system, as in pseudoplethoric subjects, who appear to have abundance of blood and muscular tissue, and yet are anæmic in extreme.

Hence, we have pale, sallow, grayish, cachectic looking individuals, with hollow cheeks and staring eyes, stoop-shouldered and gaunt, distress pictured in every motion, or stout, highly colored, and rotund, with a weakness of organism, in which every slight departure from the most ordinary routine existence —sometimes *without* that departure, and during the strictest regimen—causes functional disturbances so intense that life is a mockery, and death welcome delivery.

If we inquire still further into the distress of the victim, we find almost every known *functional disorder*, which means, eventually, in modern parlance, *organic disease*. These terms are misnomers, as there can be no chronic *functional disturbance* without *disease* of the *organ*, although there may not yet be disintegration or destruction of tissue, which would be a *lesion*.

We find dyspeptics under the strictest dietetic and hygienic rules upon whom the most wholesome food acts as poison, and the most unwholesome most unexpectedly agrees; who alternately starve and feast themselves with the same deplorable results.

Long standing constipations; hemorrhoids; distressing headaches and menstrual difficulties; annorexia and canine hunger; palpitations and night-sweats; bilious attacks; coughs and frequent colds; severe kidney complications; disturbed mental equilibrium; weakened mental faculties; forgetfulness; aphasia; paralytic and apoplectic seizures; urinary difficulties; chronic tendency to boils, ulcers, eruptions; abnormal secretions and discharges; perversions in quantity and quality of normal discharges,—are but few of the complications that arise, and may be clearly traced to the common origin.

We will find one patient to be heavy, fat, slow, groggy, with pallid, livid complexion, insensible to pain or suffering, slow to gather his wits together; slow to learn what is wanted of him. Another, rosy-cheeked upon the slightest excitement or pain, rotund, comfortable looking, yet weakened and anæmic in the extreme.

Again, he will be long, lean, lank, stoop-shouldered, hollow-eyed, obstinate, philosophizing upon every possible subject, and making nothing of it, or upon religious topics, which he never settles; misanthropic.

Again, rosy and fair to look upon as a young god, with a scrofulous and hemorrhagic diathesis that will slowly sap the life away, or fat, flabby, and useless.

Still another, emaciated, waxen, pallid, tall, hollow-chested, hollow-eyed, with inanition for the only substance that can restore him to health, he drags out a miserable existence.

Again, he dreads the approach of daily duties, even those to which he has been accustomed for years, yet when once aroused will go through those duties gloriously.

It may take the form of fear, fear of suicidal attempts; a continual urging from within to end his misery and constant suffering; a fear of dangerous situations or sudden temptations.

All this and much more we find developed by this hydra-headed monster, psora. Had it been correctly treated in its first manifestation, it would have remained latent with comparatively little discomfort to the organism.

Such conditions, uncomplicated by previous medication or other miasms, are comparatively easy of cure by the use of the dynamized similar remedy. The tangled threads of the web of life may again be tied, and the hand at the loom again be able to renew the broken pattern, smoothly weaving out both warp and woof to the very end.

In complicated cases the process must be longer, the choice of the remedy will be much more difficult, and the temper of both physician and patient will be repeatedly tried—yet the *problem may be solvèd.*

S. L. G. Leggett.

